# Circulating tumor DNA detectable in early- and late-stage colorectal cancer patients

**DOI:** 10.1042/BSR20180322

**Published:** 2018-07-31

**Authors:** Ying-Chi Yang, Dong Wang, Lan Jin, Hong-Wei Yao, Jing-Hui Zhang, Jin Wang, Xiao-Mu Zhao, Chun-Ying Shen, Wei Chen, Xue-Liang Wang, Rong Shi, Si-Yi Chen, Zhong-Tao Zhang

**Affiliations:** 1 Department of General Surgery, Beijing Friendship Hospital, Capital Medical University; Beijing Key Laboratory of Cancer Invasion and Metastasis Research and National Clinical Research Center for Digestive Diseases, Beijing 100050, China; 2 San Valley Biotechnology Incorporated, Beijing 100094, China; 3 Departments of Molecular Microbiology and Immunology and Norris Comprehensive Cancer Center, Keck School of Medicine, University of Southern California, Los Angeles, CA, U.S.A.

**Keywords:** CRC, ctDNA, genes, mutation

## Abstract

Characterization, diagnosis, and treatment of colorectal cancers (CRC) is difficult due to limited biopsy information, impracticality of repeated biopsies, and cancer biomarker fallibility. Circulating tumor DNA (ctDNA) has recently been investigated as a non-invasive way to gain representative gene mutations in tumors, in addition to monitoring disease progression and response to treatment. We analyzed ctDNA mutations and concentrations in 47 early- and late-stage CRC patients using a targetted sequencing approach using a panel that covers 50 cancer-related genes. ctDNA mutations in 37 genes were identified in 93.6% of the patients (*n*=47). The results showed that *TP53, PIK3CA, APC*, and *EGFR* were the most frequently mutated genes. Stage IV patients had significantly higher ctDNA concentration than Stage I patients, and increased ctDNA concentration correlated with increased tumor size. Additionally, ctDNA detection was found to be a greater predictor of disease when compared with five known commonly used tumor biomarkers. The present study supports the use of ctDNA as a liquid biopsy to gain clinical tumor information that may facilitate early diagnosis and treatment and improve CRC patient prognosis.

## Introduction

Colorectal cancer (CRC), with over 1.3 million new cases worldwide annually, can be difficult to detect at early stages due to non-specific symptoms which may lead to delayed diagnosis [1,2]. Characterization of the tumor can aid in deciding the course of treatment. However, these tumors are heterogeneous in nature and getting accurate information on the entire tumor’s characteristics through a biopsy or tumor sample can be challenging [3]. Tumor biomarkers in blood, including carcinoembryonic antigen (CEA) and carbohydrate antigen19-9 (CA19-9), have been used for detecting and monitoring tumor progression in CRC patients, and elevated levels of both these markers are associated with poor prognosis [4,5]. However, these biomarkers are not specific to a single cancer type and may be elevated due to unrelated conditions and thus are not entirely reliable [6]. As tumors are not always accessible for biopsy or repeated biopsy taken together with the fact that other biomarkers are not totally infallible, there is an urgent need to develop standardized, non-invasive techniques to characterize tumors, monitor disease progression, and response to treatment.

Recently, circulating tumor DNA (ctDNA) is being investigated as a non-invasive, easily obtainable sample to gain information, such as genetic mutations and tumor burden, in various types of tumors [7,8]. Some researchers have shown that ctDNA samples can be taken repeatedly and used to monitor changes during treatment, such as emerging *KRAS* mutations during anti-EGFR antibody treatment [9,10]. Others have also found that increasing ctDNA levels can predict residual or recurring disease [10,11]. Despite all the recent advances in clinical ctDNA research, standardized methods for ctDNA detection and sequencing are lacking. For example, some studies use PCR-based assays to analyze one or two genes, whereas others use the whole-genome or whole-exome analysis to detect genetic changes [12,13]. While these differing approaches offer valuable clinical information, both have pros and cons: targetted assays are more sensitive but may fail to identify important mutations that might influence the course of treatment; the whole-genome analysis is more comprehensive but less sensitive and less cost- and time-effective, all of which are important considerations when treating the cancer patients [14,15].

The goal of the present study was to use a highly sensitive targetted sequencing approach to identify ctDNA mutations in a panel of 50 cancer-related genes in 47 early- and late-stage CRC patients. Additionally, we compared ctDNA concentrations with other known cancer biomarkers mutations to analyze if ctDNA is a better predictor for disease, and ctDNA concentrations were compared amongst patients of different cancer stages to investigate, if these factors were correlated.

## Materials and methods

### Ethics statement and patients

This prospective study with 47 CRC patients was approved by the Department of General Surgery, Beijing Friendship Hospital. All the patients provided appropriate written informed consent for the use of blood under the approval of the Ethics Committee of Department of General Surgery, Beijing Friendship Hospital. All samples and medical data used in the present study were irreversibly anonymized. Blood samples were collected from each patient during surgical tumor resection and traditional serum tumor markers (CEA, AFP, CA19-9, CA125, and CA72-4) were measured at the same time.

### DNA sample preparation

EDTA tubes containing blood samples were centrifuged for 10 min at 1600 ***g***. Supernatants were further centrifuged for 10 min at 6000 ***g*** and plasma was collected and stored at −80°C until further use. At least 1 ml plasma was used to extract ctDNA with the QIAamp Circulating Nucleic Acid kit (QIAGEN, Germantown, MD) following the manufacturer’s instructions. DNA was quantitated with the Qubit 2.0 Fluorometer and the Qubit dsDNA HS Assay kit (Life Technologies, Carlsbad, CA) using the recommended protocol. ctDNA was sequenced using the SV-CA50-ctDNA panel of 50 genes frequently mutated in CRC to identify candidate mutations for ctDNA analysis. Patients with solid tumors are enrolled in the group’s San Valley database. The SV-CA50-ctDNA panel allows simultaneous detection of hundreds of variants across 50 genes relevant to solid tumors and includes solid tumor genes targetted by on-market oncology drugs and a published evidence, which includes content aligned to approved therapies and indications [16].

### Ion PGM library preparation, sequencing, and variant calling

Ion proton library preparation and DNA sequencing was performed as described in our previous studies [16–18]. An adapter-ligated library was prepared with the Ion AmpliSeq Library Kit 2.0 following manufacturer’s instructions (Life Technologies). Briefly, 10–20 ng pooled amplicons were end-repaired and ligated to Ion Adapters X and P1. Adapter-ligated products were purified with AMPure beads (Beckman Coulter, Brea, CA), nick-translated, and amplified for five PCR cycles. After purification with AMPure beads, the library concentration and size were verified with the Agilent 2100 Bioanalyzer and Agilent Bioanalyzer DNA High-Sensitivity LabChip (Agilent Technologies), respectively.

The Ion OneTouch system and the Ion PI Template OT2 200 Kit v3 (Life Technologies) were used for sample emulsion PCR and emulsion breaking, per the manufacturer’s instructions. After recovering ion sphere particles (ISPs), Dynabeads MyOne Streptavidin C1 beads on the Ion One Touch ES (enrichment system) (Life Technologies) were used to enrich template-positive ISPs, which was confirmed using the Qubit 2.0 Fluorometer. Finally, the Ion PI Sequencing 200 Kit v3 (Life Technologies) was used for sequencing barcoded samples on Ion PI v2 Chips for 100 cycles on the ion proton, as per the recommended protocol.

The SV-CA50-ctDNA panel (San Valley Biotech Inc., Beijing, China) was used to detect somatic mutations in the following 50 cancer-related genes: *ABL1, AKT1, ALK, APC, ATM, BRAF, CDH1, CDKN2A, CSF1R, CTNNB1, EGFR, ERBB2, ERBB4, EZH2, FBXW7, FGFR1, FGFR2, FGFR3, FLT3, GNA11, GNAQ, GNAS, HNF1A, HRAS, IDH1, IDH2, JAK2, JAK3, KDR, KIT, KRAS, MET, MLH1, MPL, NOTCH1, NPM1, NRAS, PDGFRA, PIK3CA, PTEN, PTPN11, RB1, RET, SMAD4, SMARCB1, SMO, SRC, STK11, TP53*, and *VHL*. This 50-gene panel and 2856-associated mutational hotspot loci was selected based on the Chinese cancer sequence database (San Valley Inc.) that contains sequencing data from more than 30000 cancer patients and 13 different cancer types.

The ion proton platform-specific pipeline software Torrent Suite was used to process initial sequencing data to generate sequence reads, trim adapter sequences, and filter and remove poor signal-profile reads. Additionally, the Torrent Suite Software with a variant ‘caller v4.0’ plug-in was used to generate initial variant calling, followed by three filtering steps to eliminate faulty base calling and generate final variant calling. First, the following definitions were made: average total coverage depth >10000; each variant coverage >10; variant frequency of each sample >0.1%; and *P -*value <0.01. Next, mutations were examined visually using integrative genomics viewer (IGV) software (http://www.broadinstitute.org/igv) or SAMtools software (http://samtools.sourceforge.net) and possible DNA strand-specific errors were removed. Finally, variants were set within the 2856-defined mutational hotspot loci.

### Tumor biomarker analysis

In addition to mutation analysis, plasma samples were analyzed for the tumor biomarkers CEA, AFP, CA19-9, CA125, and CA72-4 using an electrochemiluminescence immunoassay (Cobas E601; Roche, Indianapolis, IN, U.S.A.), following the manufacturer’s instructions. The following cut-off values were used to identify samples with elevated biomarker expression: CEA >5.0 ng/ml, AFP >5.4 ng/ml, CA19-9 >37.0 U/ml, CA125 >35.0 U/ml, and CA72–4 >5.3 U/ml. All the measurements were performed in duplicate.

### Statistical analysis

Fisher’s exact test was used to calculate *P*-values using GraphPad QuickCalcs Online Calculator for Scientists (http://www.graphpad.com/quickcalcs). All *P-*values are two-tailed and statistical significance was defined as *P*<0.05.

## Results

### ctDNA mutations identified by targetted sequencing

**Figure 1:**
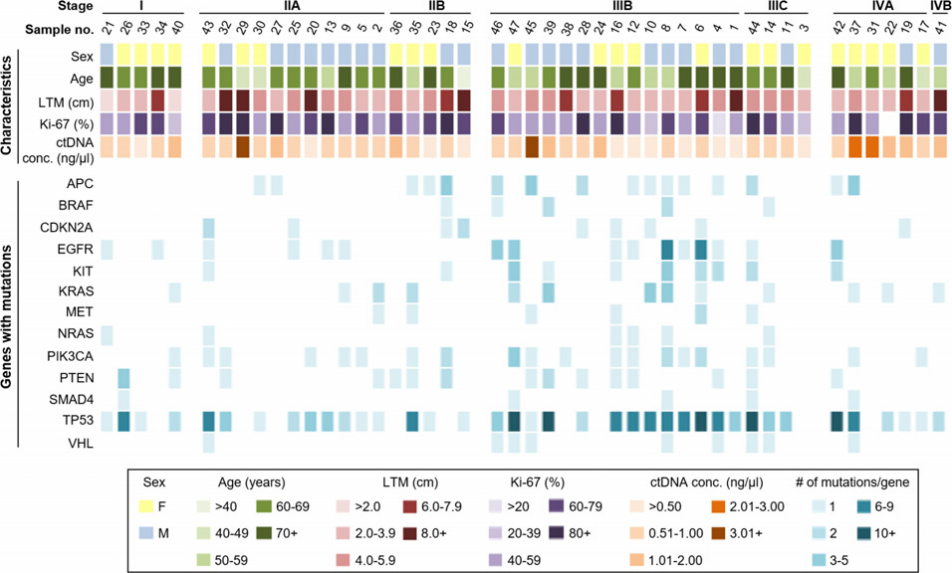
Summary of patient characteristics and gene mutations in ctDNA samples Patients were categorized based on stage, sex, age, longest tumor measurement (LTM), Ki-67 status (%), and ctDNA concentration (top), and number of mutations per gene (bottom).

Forty-seven CRC patients were enrolled in the study, the clinical features of them were represented in Table 1. Plasma ctDNA samples obtained from CRC patients during surgery were analyzed for mutations in a panel of 50 CRC-related genes. Mutations were identified in 39/50 (78%) of the genes sequenced; however, only 13 genes contained mutations in 10% or more of all patients. Mutations were most commonly identified in *TP53, EGFR, APC, PIK3CA*, and *KRAS* genes (43.0, 7.1, 5.8, 5.4, and 4.7% of all mutations, respectively), and in other genes but at lower frequencies. The number of mutations in these genes per patient, along with patient characteristics, is illustrated in Figure 1.

**Table 1:** Clinical features of 47 CRC patients

Characteristic	Parameter value
Age, years	
Mean (S.D.)	63 (10.64)
Median (range)	63 (36–80)
Sex	
Male	24
Female	23
Tumor type	41
Ulcerated	39
Elevated	8
Tumor stage	
I	5
IIA	11
IIB	5
IIIB	15
IIIC	4
IVA	6
IVB	1

Amongst all patient samples examined in the present study, we found that the average number of mutated genes per patient was 5.0 (S.D. ±3.9, range: 0–14), and the average number of total mutations per patient was 9.9 (S.D. ±9.9, range: 0–41). Three patients (at the Stages of IIIB, IIA and IVA, respectively) had no mutations in the 50 genes screened and seven patients had only one gene with mutation(s). Remaining 37 patients had two or more genes with mutations (average: 6.2 mutated genes per patient, S.D. ± 3.6, range: 2–14) and the average number of mutations per patient in this group was 12.3 (S.D. ± 9.8, range: 2–41). The number of mutated genes and total number of mutations varied slightly between patients of different stages: Stage I had an average of 3.0 mutated genes and 5.0 total mutations per patient; Stage II averaged 4.1 mutated genes and 6.2 mutations per patient; Stage III averaged 6.7 mutated genes and 15.1 mutations per patient; and Stage IV patients had an average of 4.1 mutated genes and 8.0 mutations per patient.

The most common gene with mutations found in 38/47 (80.9%) of the patients was *TP53*. The other commonly mutated genes, *PIK3CA, APC, EGFR, and KRAS*, were found in 20/47 (42.6%), 16/47 (34.0%), 16/47 (34.0%), and 13/47 (27.7%) of patients, respectively. Approximately 70% of the patients (33/47) had multiple mutations within a single gene, most commonly in *TP53* and *EGFR*. Interestingly, patients with the highest ctDNA concentrations (over 1.0 ng/μl) had fewer mutated genes and nearly half as many mutations per patient when compared with patients with lower ctDNA concentrations (3.1 compared with 5.7 mutated genes/patient and 6.3 compared with 11.1 mutations/patient, respectively).

### ctDNA concentration and tumor characteristics

**Figure 2:**
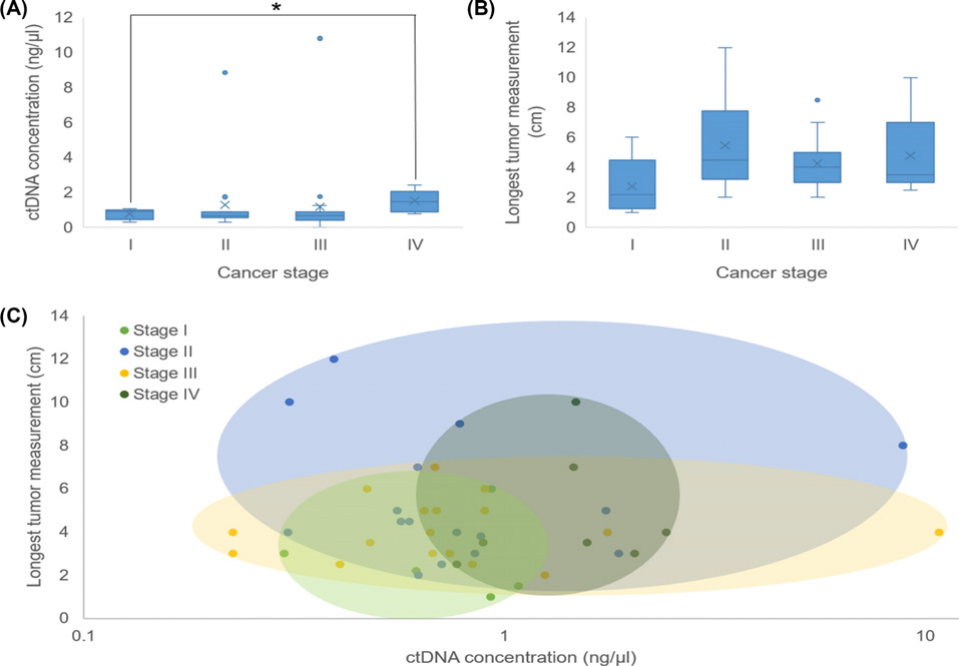
Comparison of ctDNA concentration, tumor size, and cancer stage (**A**) Box and whisker plot of patients’ ctDNA concentration compared with cancer stages. The ctDNA concentration in patients with Stage I cancer was significantly less than that in patients with Stage IV cancer (*P* = 0.0149). (**B**) Box and whisker plot of each patient’s LTM (cm) compared with cancer size. (**C**) Colored ovals represent the range of ctDNA concentration compared with LTM for each patient based on cancer stage.

The ctDNA concentration was measured for each patient and compared with the respective patient’s tumor characteristics, including the stage and longest tumor measurement (LTM). Results showed that the concentration of ctDNA increased with cancer stage and a significant difference was observed in the ctDNA concentration from Stage I patients compared with Stage IV patients (*P*=0.0149) (Figure 2A). Figure 2B shows the LTM for each patient by cancer stage and although no significant differences were observed between the cancer stages, the tumor size appeared to increase with cancer stage. We then compared ctDNA concentration and LTM based on cancer stage. The colored ovals in Figure 2C correspond to the cancer stage and show the range of ctDNA concentration and LTM for each stage. Stage III tumors varied the least in size but these patients had the widest range of ctDNA concentrations measured amongst all patients, whereas Stage II tumors varied the most in size and also had a wide range of ctDNA concentrations. Stage I and Stage IV tumors varied the least in size and patient ctDNA concentration, but stage IV cancers were on an average bigger and ctDNA concentrations were higher. Regardless of stage, the average ctDNA concentration for patients with tumors >3 cm was 0.722 ng/μl. Patients with tumor size 3–6 cm had an average ctDNA concentration of 1.27 ng/μl while for tumors <7 cm, the average ctDNA concentration was 1.62 ng/μl. Though not statistically significant, we observed a trend where higher ctDNA concentration correlated with increased tumor size.

### ctDNA concentration compared with other tumor biomarkers

**Figure 3:**
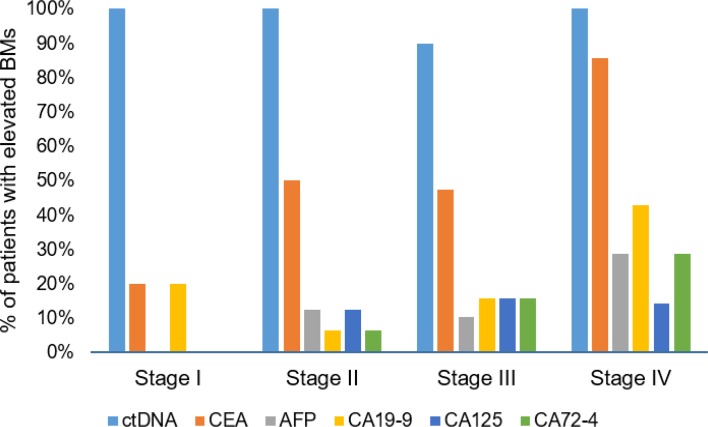
Comparison of detectible ctDNA compared with other cancer biomarkers ctDNA was detectible in nearly all patients regardless of cancer stages, whereas fewer cancer biomarkers were detectible in patients with early stage cancers.

In addition to measuring the ctDNA concentration for each patient, we also measured the concentration of several known tumor biomarkers such as CEA, AFP, CA19-9, CA125, and CA72-4. Number of patients with one or more elevated biomarkers increased with cancer stage: 20.0 in Stage I, 68.8 in Stage II, 84.2 in Stage III, and 100.0% in Stage IV patients. Figure 3 shows that relatively few patients with early stage cancer had elevated biomarker concentrations compared with those with later stage cancers. ctDNA, on the other hand, was above the lower detection limit for nearly all patients, regardless of cancer stage. Thus, ctDNA had a higher detection rate than then tumor biomarkers tested in the present study suggesting that ctDNA is a better indicator of disease.

## Discussion

The present study analyzed plasma ctDNA in 47 early- and late-stage CRC patients using targetted sequencing to identify mutations in a defined panel of 50 known cancer-related genes and oncogenes. We found that 93.6% of patients, including all Stage I patients had detectable levels of ctDNA and mutations were identified in 39/50 (78%) genes in the cancer panel. Most patients (80.9%) had multiple mutations within a single gene and/or across multiple genes. Our mutation analysis revealed that *TP53* was mutated in 43/47 (80.9%) of patients, and mutations were also frequently detected in *EGFR, APC, PIK3CA*, and *KRAS*. One potential advantage of the present study over others is that compared with PCR-based assays to analyze one or two genes, the SV-CA50-ctDNA panel contain the important mutations about CRC treatment sensitively; hence removing the bias present in PCR-based analyses of a few genes.

Many of the mutated genes in our study are significant in a clinical setting and would potentially influence drug treatments. Alterations in genes involved in the pathways downstream of EGFR activation, such as *KRAS, NRAS, BRAF*, and *PIK3CA*, may lead to drug resistance due to constitutive EGFR activation [19]. Xu et al. [20] reported that *PIK3CA* mutations identified in ctDNA were found to contribute to acquired cetuximab resistance in patients with metastatic CRC. Recent studies have monitored acquired *KRAS* mutations in ctDNA during anti-EGFR antibody treatment of advanced CRC patients. These mutations, which render certain drugs ineffective, were detectable in ctDNA prior to radiographic detection of disease progression and decreased when treatment was discontinued [9,21]. A recent meta-analysis found that ctDNA provides a higher diagnostic accuracy (95%) for *KRAS* mutations in CRC patients [22]. We found that 36.2% (17/47) of patients had two or more combination mutations in *PIK3CA, EGFR, KRAS, NRAS*, and *BRAF*. Because of their implications in treatment, a targetted approach that can identify ctDNA mutations in several key genes simultaneously would aid in treatment and potentially improve patient outcome.

Consistent with the previous studies showing that ctDNA levels correspond to tumor burden [23,24], we found that overall, the concentration of ctDNA increased with tumor size. ctDNA concentrations were also found to correlate with the cancer stage, where Stage IV patients had a significantly higher ctDNA than those with Stage I cancer (1.52 compared with 0.77 ng/μl, respectively). Interestingly, while Stage IV patients had the highest concentration of ctDNA, this group was only slightly over one mutated gene per patient than those with Stage I CRC. Also of note, two Stage IV patients had a relatively low-ctDNA levels (>1.0 ng/μl) and relatively small tumors (LTM of 2.5 and 3.5 cm). These results are in line with the study of Morbelli et al. [25] who found that in advanced lung cancer patients, in addition to tumor burden, ctDNA also reflected tumor aggressiveness. Thus, measuring ctDNA levels may provide important prognostic information. Bettegowda et al. [7] reported that metastatic CRC patients who had a lower ctDNA levels lived significantly longer compared with those with higher ctDNA levels. Our study has a limitation as we did not follow-up with patients after surgery to check if ctDNA levels or mutations correlated with survival. Future large-scale studies on ctDNA and prognosis/survival are warranted, especially in patients with early stage cancers.

We also compared ctDNA concentration with the expression of five clinically relevant biomarkers, including CEA, CA19-9, CA125, CA72-4, and AFP. These biomarkers have been found to be expressed at higher levels in CRC patients prior to surgery and upon disease recurrence, and are associated with more advanced disease [26]. AFP has also been used to evaluate the effect of surgical resection in CRC patients [27]. CEA is the most utilized biomarker for monitoring CRC where elevated levels indicate poor prognosis; however, elevated CEA levels may also be due to unrelated conditions [28]. We found that the number of patients with one or more elevated biomarker increased with cancer stage, where CEA was elevated in 20% of Stage I patients compared with 86% of Stage IV patients. In comparison, we detected ctDNA above the limit of 0.01 ng/μl in nearly 96% of the patients, including all Stage I patients. Early detection of cancer is paramount to successful treatment, including early detection of recurrence after surgery. Two recent studies found that monitoring ctDNA levels in CRC patients provide an earlier indication of disease recurrence and response to treatment in comparison with CEA or radiological diagnosis [23,29]. It is possible that the result of ctDNA detection in all Stage I patients in our study was due to the small sample size. Further research with early stage patients is necessary to see if ctDNA is a useful biomarker in early diagnosis.

In conclusion, the present study supports the use of targetted sequencing to identify mutations in ctDNA from early- and late-stage CRC patients. Our study is clinically significant as cancer patients may benefit from ctDNA analysis as it is a relatively easy and a non-invasive way to supplement traditional diagnostics with real-time tumor information and exhibit a much greater sensitivity than other tumor biomarkers. Further research with larger groups of patients is required before standardized detection and sequencing methods are established.

## Abbreviations

CA19-9: carbohydrate antigen 19-9
CEA: carcinoembryonic antigen
CRC: colorectal cancer
ctDNA: circulating tumor DNA
IGV: integrative genomics viewer
ISP: ion sphere particle
LTM: longest tumor measurement
